# Crown Fragment Reattachment by the Minimal Intervention Approach for Coronal Tooth Fractures: A Case Series

**DOI:** 10.7759/cureus.48710

**Published:** 2023-11-12

**Authors:** Kanav Jain, Lata Goyal, Padmanidhi Agarwal

**Affiliations:** 1 Conservative Dentistry, Chandra Dental College, Lucknow, IND; 2 Dentistry, All India Institute of Medical Sciences (AIIMS) Bathinda, Bathinda, IND; 3 Dentistry, Dr. Ram Manohar Lohia Institute of Medical Sciences, Lucknow, IND

**Keywords:** tooth crown, reattachment, incisor, permanent dentition, esthetics, crowns, tooth fracture

## Abstract

Dental trauma is the most frequently encountered injury that requires immediate attention. Several procedures are available to manage broken teeth afflicted by trauma but the choice of procedure depends upon structural, functional, and esthetic considerations. The goal is to choose the least invasive, immediate, and simple technique that can meet the patient’s expectations. The aim of this case series is to present a minimal intervention approach as a primary treatment option for the reattachment of trauma-induced fractured coronal tooth fragments, to preserve and enhance function, esthetics, and structure.

## Introduction

Fracture of the coronal part of anterior teeth is the most common injury in the oral cavity in permanent dentition, affecting both children and adolescents [1]. The most common tooth fracture is of the maxillary central incisor due to its proclined angulation and front position in the dental arch. Mandibular teeth are the least affected [2].

These crown fractures require immediate attention and when left untreated can have structural, functional, and psychological impacts on the patient [1]. Several factors should be considered while managing these like the extent of the fracture line, vitality of involved teeth, location of fracture line (supra- or sub-gingival), involvement of periodontium, invasion of biological width, soft tissue trauma, preservation of the fractured segment and current condition, approximation of intact teeth with fractured segment, occlusion, prognosis [3]. A patient’s ability to grasp the situation, treatment, and prognosis and cooperate for the same is very important for long-term outcomes.

To manage such injuries traditionally, treatment options like laminates, composite restoration, post and core, surgical repositioning of the segment by orthodontic extrusion, and periodontal crown lengthening can be considered. In the end, all these techniques turned out to be time-consuming, expensive, and esthetically less satisfying [4].

If the fractured segment is available and in the right condition for preservation, reattachment of the fractured tooth fragment should be considered as the most favorable treatment modality because it is immediate and conservative. This procedure provides a simple, viable, cost-effective, and time-saving alternative [5,6].

## Case presentation

Case 1

**Figure 1:**
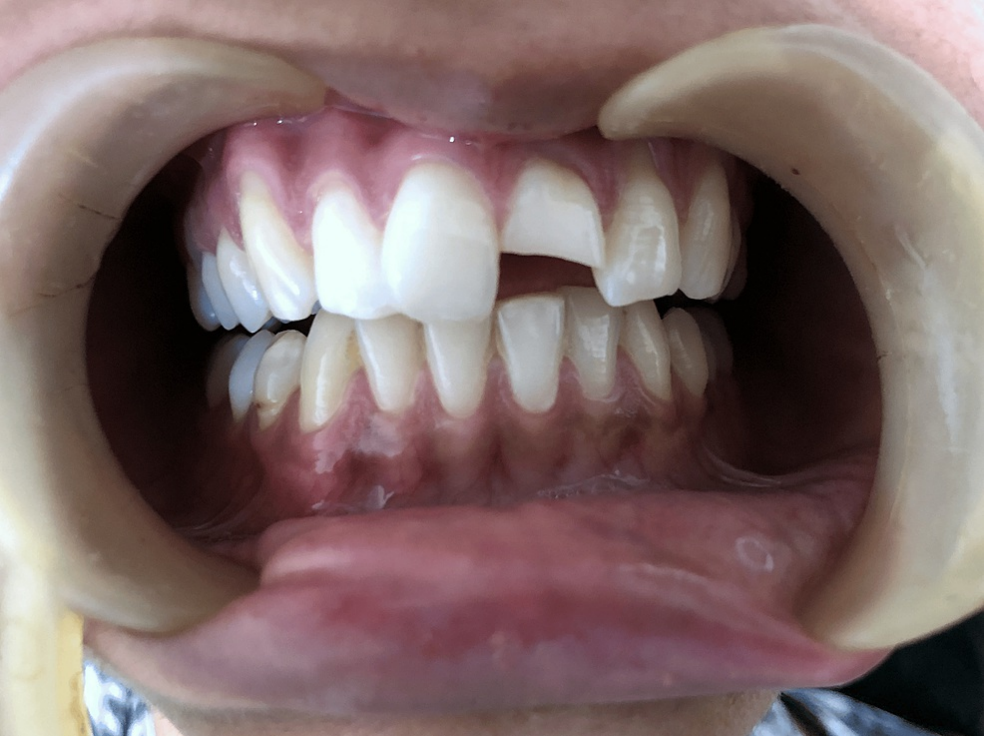
Pr operative Ellis class 3 fracture wrt 21

A 22-year-old female reported with a fractured upper front tooth, injured from a bump on the side of the bed three days prior (Figure 1).

**Figure 2:**
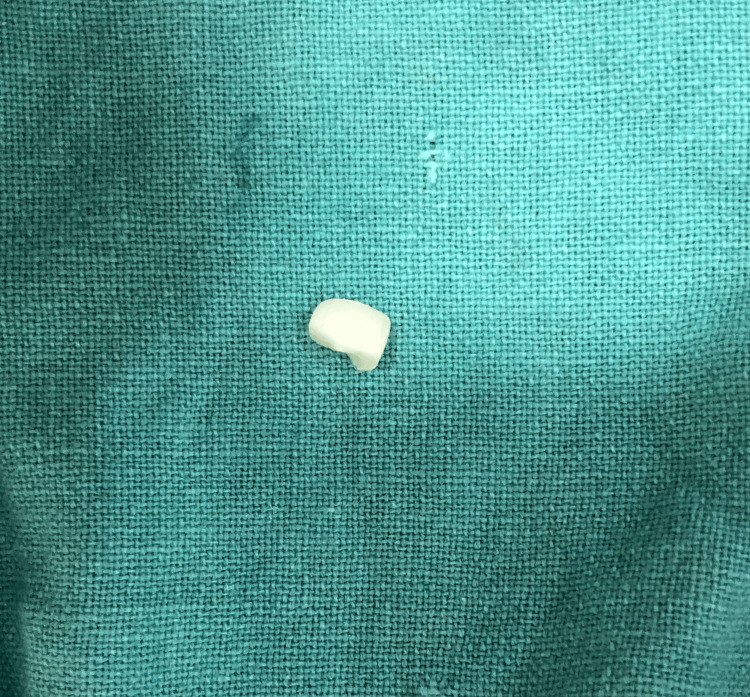
Fractured fragment

She gave a history of mild pain with no bleeding from the tooth at the time of injury. On examination, an Ellis class 3 fracture was diagnosed in 21 with the fracture line running mesiodistally in the middle third and tenderness on percussion. The patient had stored the broken fragment in a glass of water (Figure 2).

**Figure 3:**
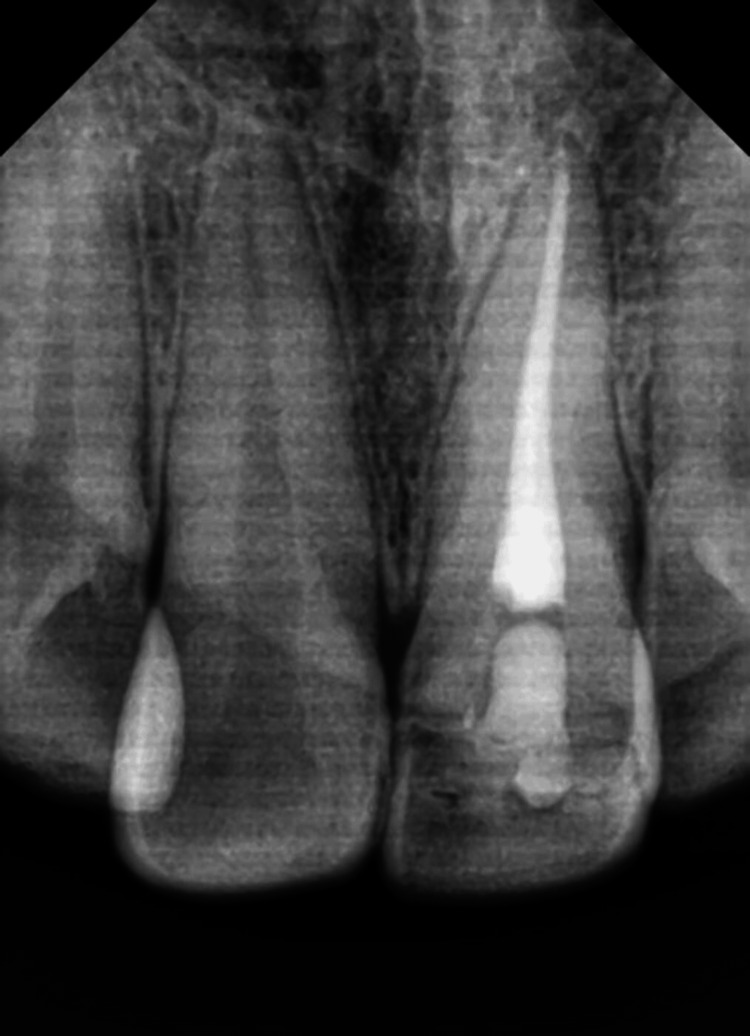
Postoperative radiograph with grooves made in the fragment

Radiographically the fracture line involved the pulp. The fragment was transferred to normal saline, and root canal treatment was initiated. Working length was taken and biomechanical preparation was performed using hand K files and rotary Hyflex files. Copious irrigation with sodium hypochlorite and normal saline was done and a temporary filling was placed. One week later, obturation was carried out by the lateral condensation technique (Figure 3).

**Figure 4:**
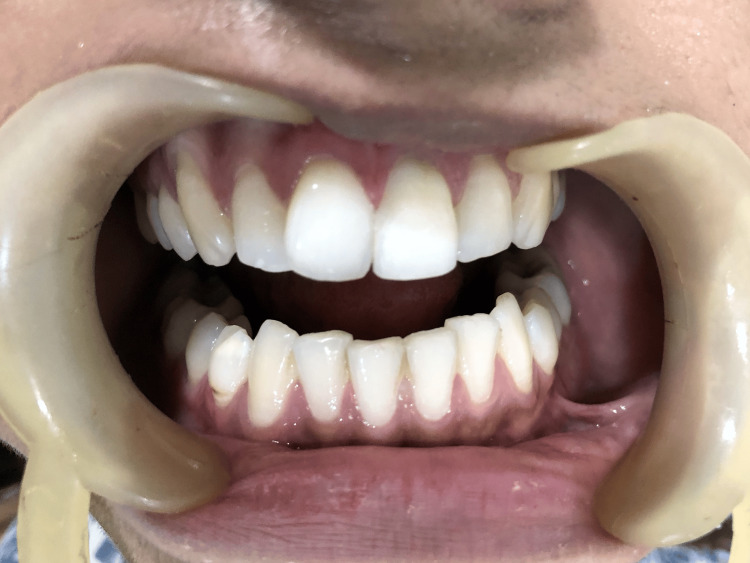
Postoperative picture with composite restoration

The tooth and the fragment were prepared with a 4 mm x 2 mm x 2 mm groove on the fractured fragment for better retention along with a facial bevel for both retention and esthetics. The tooth was then reattached with composite (Figure 4).

The patient has been on regular follow-up for over two years and has been asymptomatic since his treatment.

Case 2

**Figure 5:**
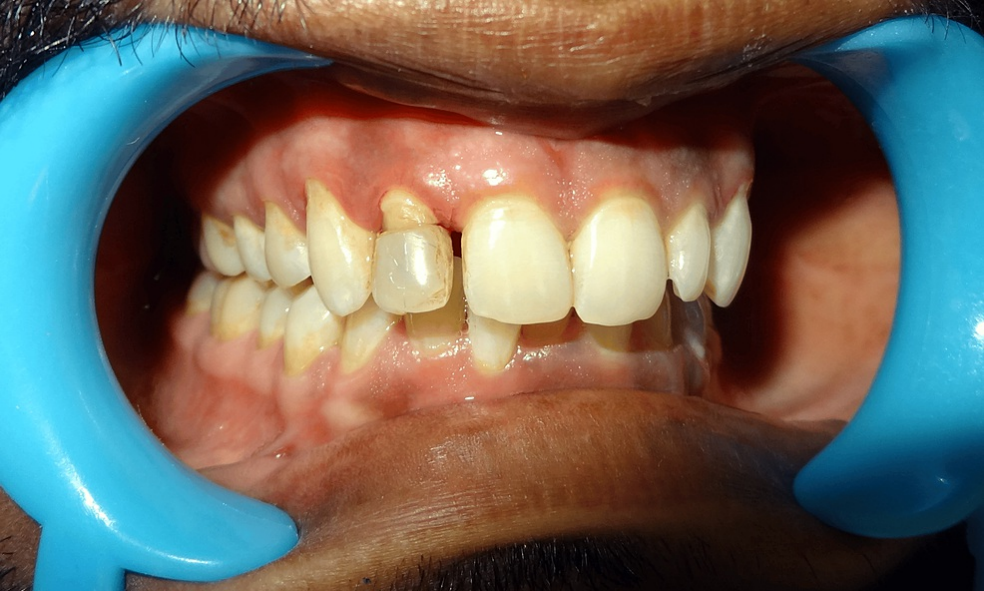
Crown-en-mass fracture wrt 12

A 35-year-old man reported with severe pain and difficulty in biting, with intermittent bleeding from the right upper front tooth for two days, when he had tripped and fallen on the stairs. On examination, a crown-en-mass fracture was observed in 12, with the palatal one-third of the crown still intact (Figure 5).

**Figure 6:**
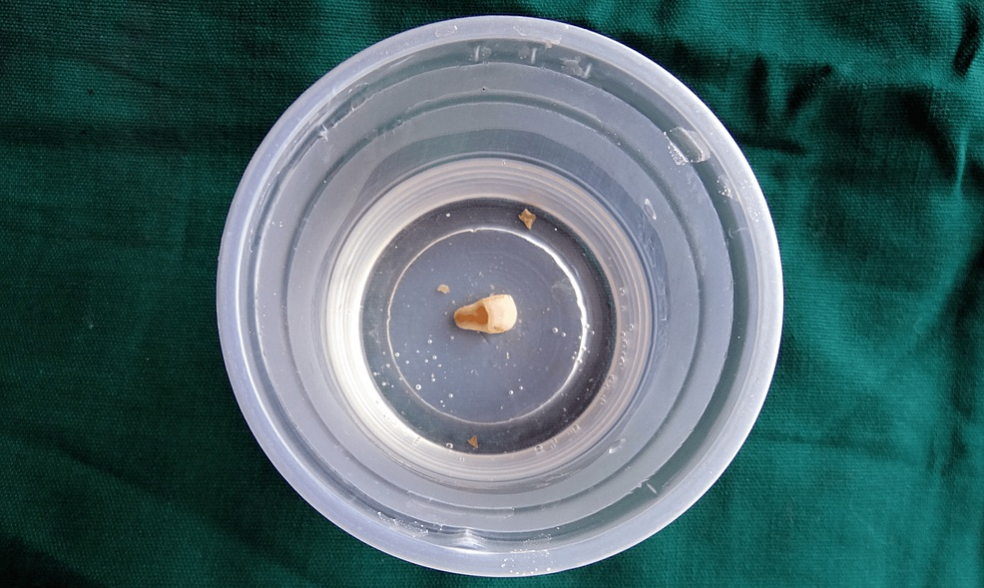
Fractured crown fragment immersed in saline

Facially, the fracture line extended to the gingival level. Percussion and palpation tests were done to rule out any dentoalveolar fracture. Radiographs were taken to rule out a root fracture. Access was not done through the broken fragment to maintain its integrity (Figure 6).

**Figure 7:**
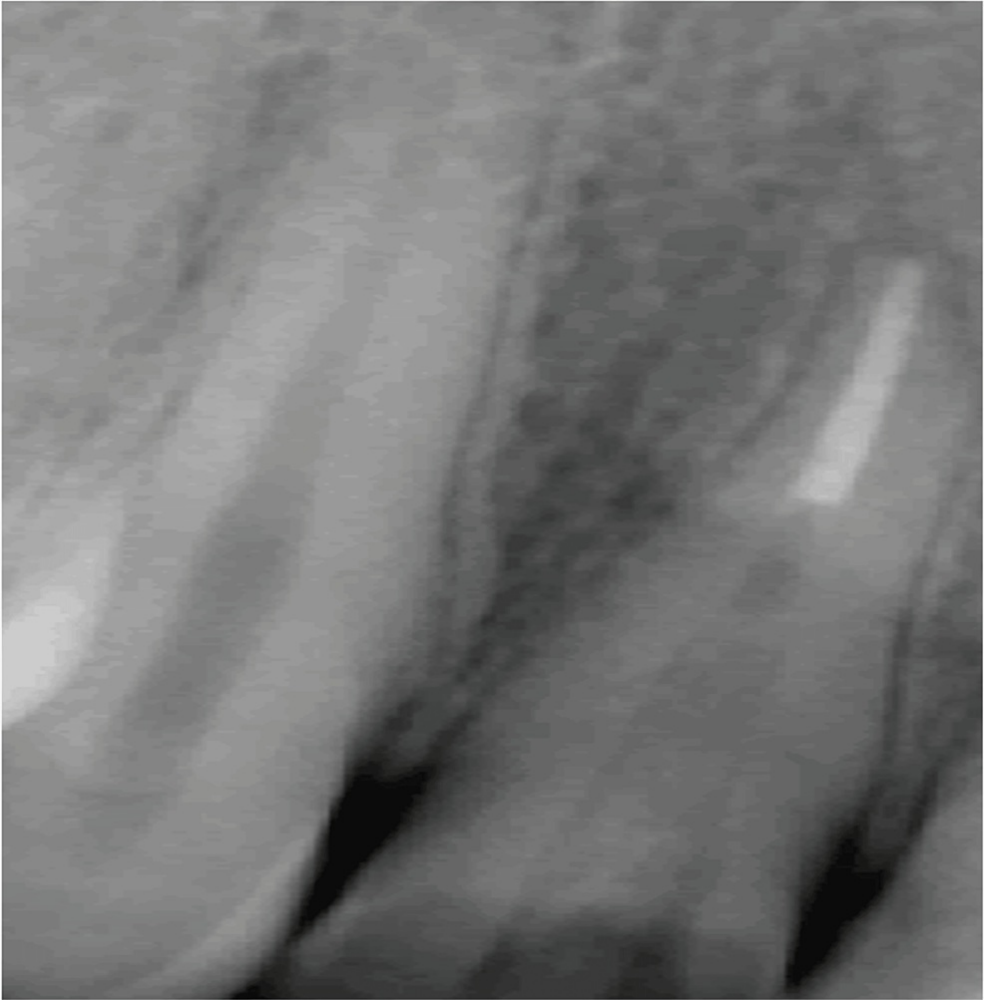
RCT treated 12 with prepared post space RCT: root canal treatment

An immediate access opening in 12 was then performed through the remaining tooth structure. Working length was taken and biomechanical preparation was done using hand K files and rotary hyflex files and obturation was completed. Post space was prepared in the root canal (Figure 7).

**Figure 8:**
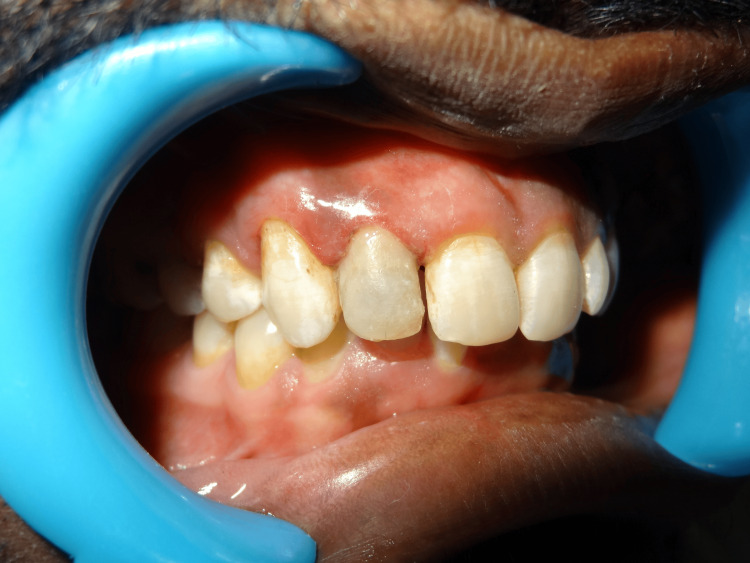
Postoperative composite restoration

A 3 mm hole was made in the fragment to accommodate it. Paracore luting cement was used to lute the post and join the fracture line. Finishing and polishing were done using shofu disks (Figure 8).

The patient has been symptom-free for the past three years and is on regular follow-up.

Case 3

**Figure 9:**
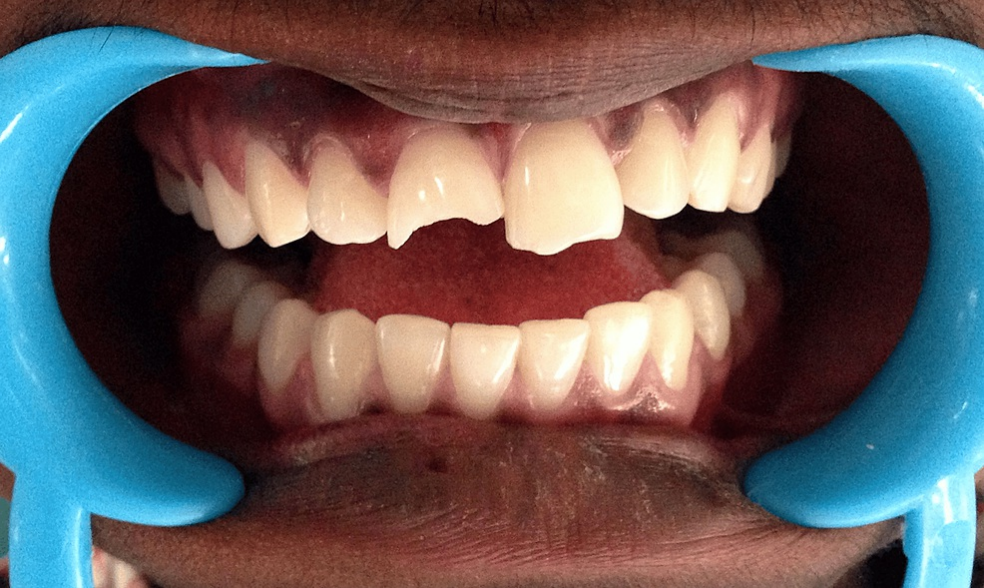
Preoperative Ellis class 2 fracture

An 18-year-old boy reported with a fractured upper front tooth while playing football a few hours ago. Examination revealed an Ellis class 2 fracture running mesiodistally from the middle third of the tooth in relation to 11 (Figure 9).

**Figure 10:**
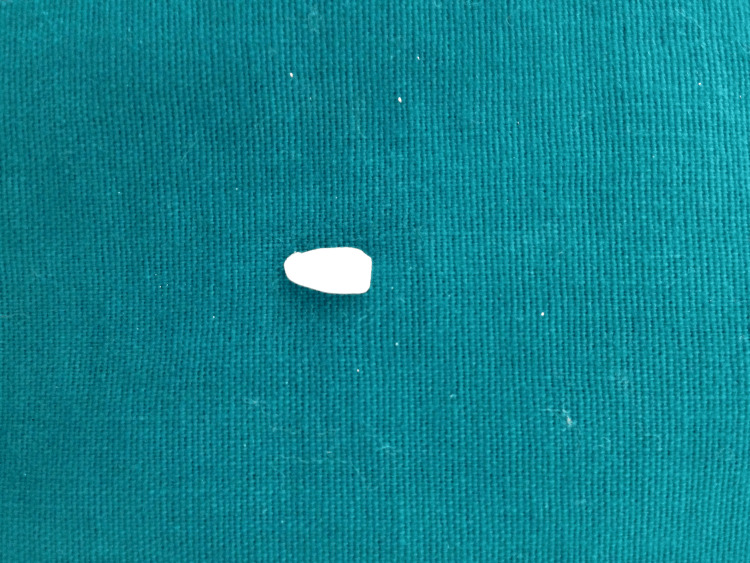
Fractured fragment

The tooth was mildly tender on percussion. The patient had recovered the fractured tooth fragment from the site of trauma and wrapped it in a handkerchief. On reporting, the fragment was immediately stored in normal saline (Figure 10).

**Figure 11:**
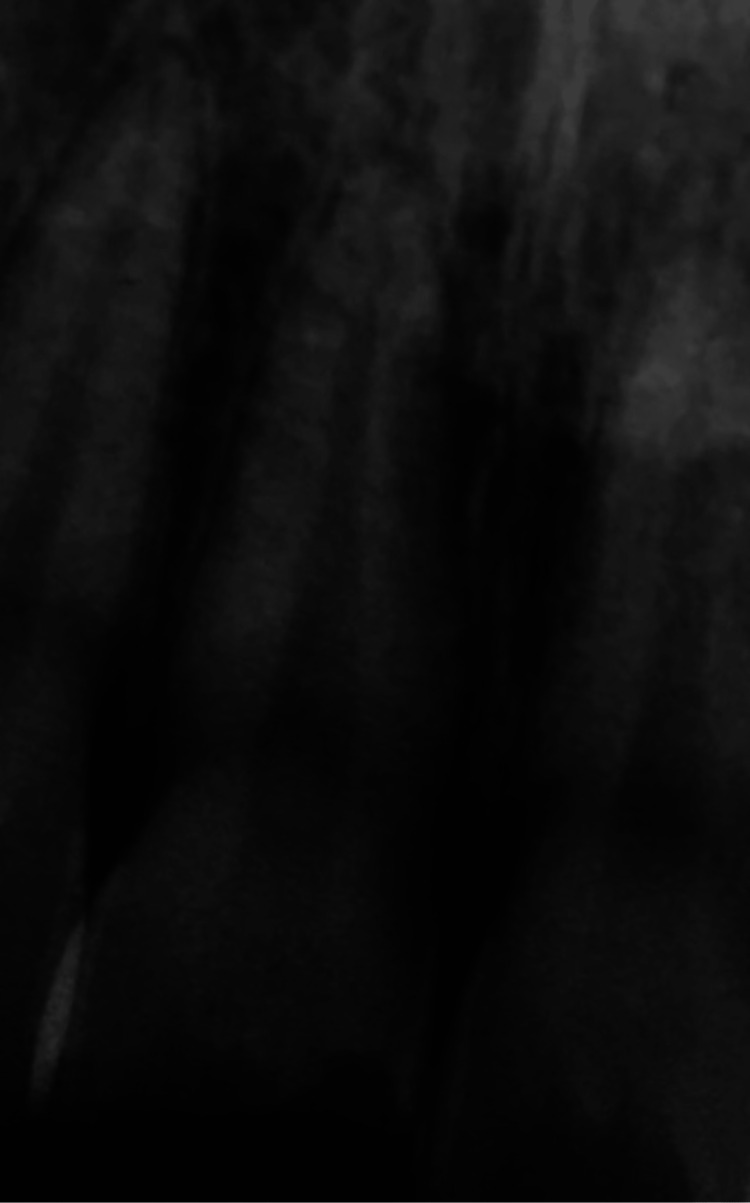
Radiograph of fractured 11 to rule out a root fracture

A periapical radiograph was taken to rule out any root fracture (Figure 11).

**Figure 12:**
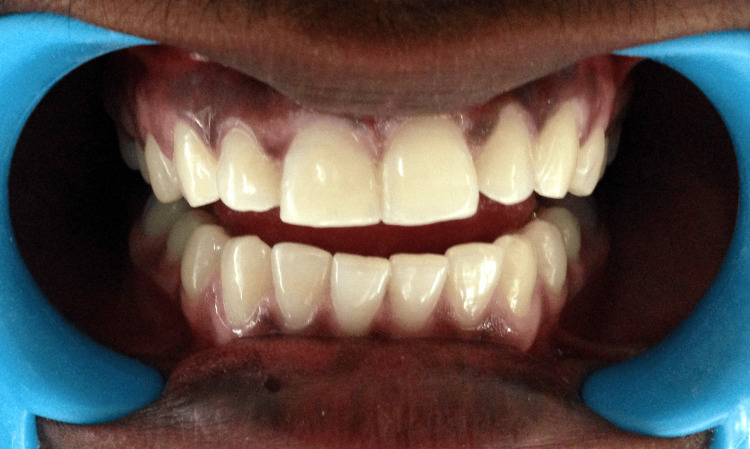
Postoperative composite restoration

The fractured fragment fit perfectly on the tooth and since the patient was worried about his aesthetics, immediate fragment reattachment was planned. A knife-edge bevel was prepared both on the tooth and the fragment and re-attached with the help of a composite (Figure 12).

The patient has been asymptomatic for 18 months.

All three patients were informed that their demographic data, clinical photographs, and radiographs would be used for academic purposes and written informed consent was obtained from them for the same.

## Discussion

This case series describes crown fragment reattachment and its benefits as a minimal interventional approach for the management of coronal tooth fractures. This technique offers several advantages, as it is less time-consuming, economical, conservative, and esthetically more predictable. The modulus of elasticity, wear, and abrasiveness of fractured fragments is similar to intact teeth in terms of shade, contour, texture, and fracture resistance [2].

Anterior maxillary teeth are the most common ones to be injured. Various treatment modalities could be opted for to manage the fractured tooth, including composite restoration, orthodontic extrusion, post and core prosthesis, and extraction followed by a prosthesis depending upon the extent of the fracture, time-lapse, root morphology, the vitality of the tooth, and the location of the fracture. The real challenge is choosing the most appropriate treatment based on evidence and clinical expertise. Loss of the coronal portion of a tooth can have a psychological impact along with obvious esthetic and functional deformities. If it is feasible, tooth fragment reattachment can be considered as it is an immediate, conservative, and most practical option to save such uncomplicated crown fractures [5].

Crown reattachment can be performed using various methods according to the site of the fracture line, the vitality of the involved tooth, and the size of the segment available. This can be seen in the differences in the method used in all three cases. Studies support the fact that the placement of an internal groove restores 90.5 % of fracture resistance [2].

Reattachment using posts further enhances the retention of the segment. When intermediate material is used, better bond strength is achieved between tooth fragments and dentin [6].

The material used for reattachment also has an influence on the fracture resistance of the segment. Singhal found that among the various materials used, composite resin has the highest fracture resistance, whereas resin-modified glass ionomer has the least resistance [7]. In all of our cases, composite resin was used rather than resin-modified glass ionomer with a groove inside the fracture segment. Bevelling of the fracture segment was done to enhance retention as well as the bulk of the material on the beveled margin to mask the fracture line and better esthetics.

Another factor for consideration is segment hydration for better optical properties. Fractured segments should be hydrated for a minimum of 15 minutes to half an hour before bonding, to enhance the esthetics and bond strength, which was done in our cases without fail. Any color mismatch, if it still occurs, may disappear within a month to a year after reattachment of the fractured segment, due to water absorption [7].

The long-term prognosis of the reattachment depends upon the location of the fracture, periodontal condition of the tooth, root development, pulp involvement, involvement of biological width zone, and material used for restoration. The supra-gingival fracture line and no violation of biological width in our cases eliminated the need for additional surgery [8].

The highlight of this manuscript is fragment reattachment by minimal intervention using three different techniques in the cases described. The long-term successful follow-up of these is also available. We advocate crown reattachment as the preferred technique for supra-gingival crown fractures. It is important to note that case selection is very important to get the choicest outcomes. Whenever opting for this, indications and contraindications have to be weighed, as discouraging results are seen in crowns with vertical root fracture, deep bite, unfavorable occlusal relationship, and bruxism [9].

Reattachment is a simple and conservative technique that requires minimum tooth preparation, gives long-term good esthetics, and is the least time-consuming. Moreover, using tooth segments minimizes color discrepancies by providing better surface texture and contours. It is the best method to decrease the financial, physical, and emotional burden on the patient.

## Conclusions

This simple, efficient, and time-saving technique can be used as the method of choice for rehabilitating tooth fractures where the fractured segment is preserved and available. Proper case selection should be done for this minimally interventional approach to produce the most aesthetic results and exceed the patient's expectations in terms of both form and function.
